# Imaging features of adenosquamous carcinoma of the breast – A rare variant of metaplastic breast carcinoma

**DOI:** 10.1259/bjrcr.20210108

**Published:** 2022-03-09

**Authors:** Tiffany Marian Sae-Kho, Asha Bhatt, Malvika H. Solanki, Elizabeth B. Jeans, Kimberly S. Corbin, Robert T. Fazzio, Katrina N. Glazebrook

**Affiliations:** 1 Department of Breast Imaging and Intervention, Mayo Clinic, Rochester, MN, USA

## Abstract

Adenosquamous carcinoma of the breast is a rare subtype of metaplastic carcinoma, which accounts for <1% of invasive breast malignancy. Metaplastic carcinoma is usually high grade and aggressive with typically reported benign imaging features when compared to invasive ductal carcinoma. However, the adenosquamous variant is a subtype with a more favorable prognosis. Within the literature, there is limited imaging description with case studies focusing on metaplastic carcinoma. Herein, we report seven cases of the adenosquamous subtype describing the imaging findings with correlation to clinical history and pathology. The majority of patients (*n* = 6) presented with palpable breast masses. One patient was identified through screening mammography. Mammographically (*n* = 6), tumors appeared as irregular masses. Sonographically (*n* = 7), tumors appeared as irregular masses ranging from solid to mixed solid/cystic masses. On MRI (*n* = 1), one tumor appeared as an irregular rim enhancing mass. FDG PET/CT (*n* = 2) and whole-body bone scan (*n* = 1) were also available for review. The majority of tumors were low-grade (*n* = 6) with only one high-grade tumor. This case series of seven patients demonstrated predominantly suspicious imaging features despite the majority being low-grade tumors.

## Introduction

Metaplastic carcinoma accounts for <5% of all breast cancer cases and usually presents as a high-grade, aggressive malignancy.^1–5^ Adenosquamous carcinoma is a rare subtype of metaplastic breast carcinoma, typically manifesting as a low-grade malignancy with an overall better prognosis.^6,7^ Adenosquamous carcinoma of the breast was first described by Rosen in 1987.^5^ The majority of the radiology literature describes imaging features of metaplastic carcinoma in general, limiting the available imaging features of the adenosquamous carcinoma subtype to case reports and case series.^2–4^ We report seven cases of adenosquamous carcinoma with correlation to clinical presentation, surgical outcome, and pathology review.

## Study protocol

### Case selection

Following institutional review board approval, a retrospective database search was performed at our institution from January 2000 to July 2019 for cases of pathologically proven adenosquamous carcinoma of the breast. Of 10,000 total breast biopsies, 1000 were malignant. seven cases of adenosquamous carcinoma were available for review and included in our study.

### Radiology

Breast imaging features were described according to the American College of Radiology Breast Imaging Report and Data System Atlas fifth Edition lexicon. Imaging features on mammography (*n* = 7), ultrasound (*n* = 7), MRI (*n* = 1), FDG PET/CT (*n* = 2), and whole-body MDP bone scan (*n* = 1) were assessed.

### Pathology

Available pathology slides were independently reviewed by a dedicated breast pathologist (M.H.S.) to confirm the diagnosis of adenosquamous carcinoma.

## Results

### Clinical presentation

Adenosquamous carcinoma typically presents as a palpable mass similar to other types of metaplastic carcinomas.^5,7^ The majority of our patients presented as a palpable mass (*n* = 6). One patient concurrently presented with nipple discharge and one patient was identified only through screening mammography. Patient age ranged from 47 to 83 years, with a mean age of 67 at diagnosis, which is in keeping with the medical literature as most reported metaplastic carcinomas are in females over the age of 50.^4,5,7,8^ To our knowledge, all previous cases have been reported in females and our seven cases involved only females.

### Imaging features

Mammography

**Figure 1.**
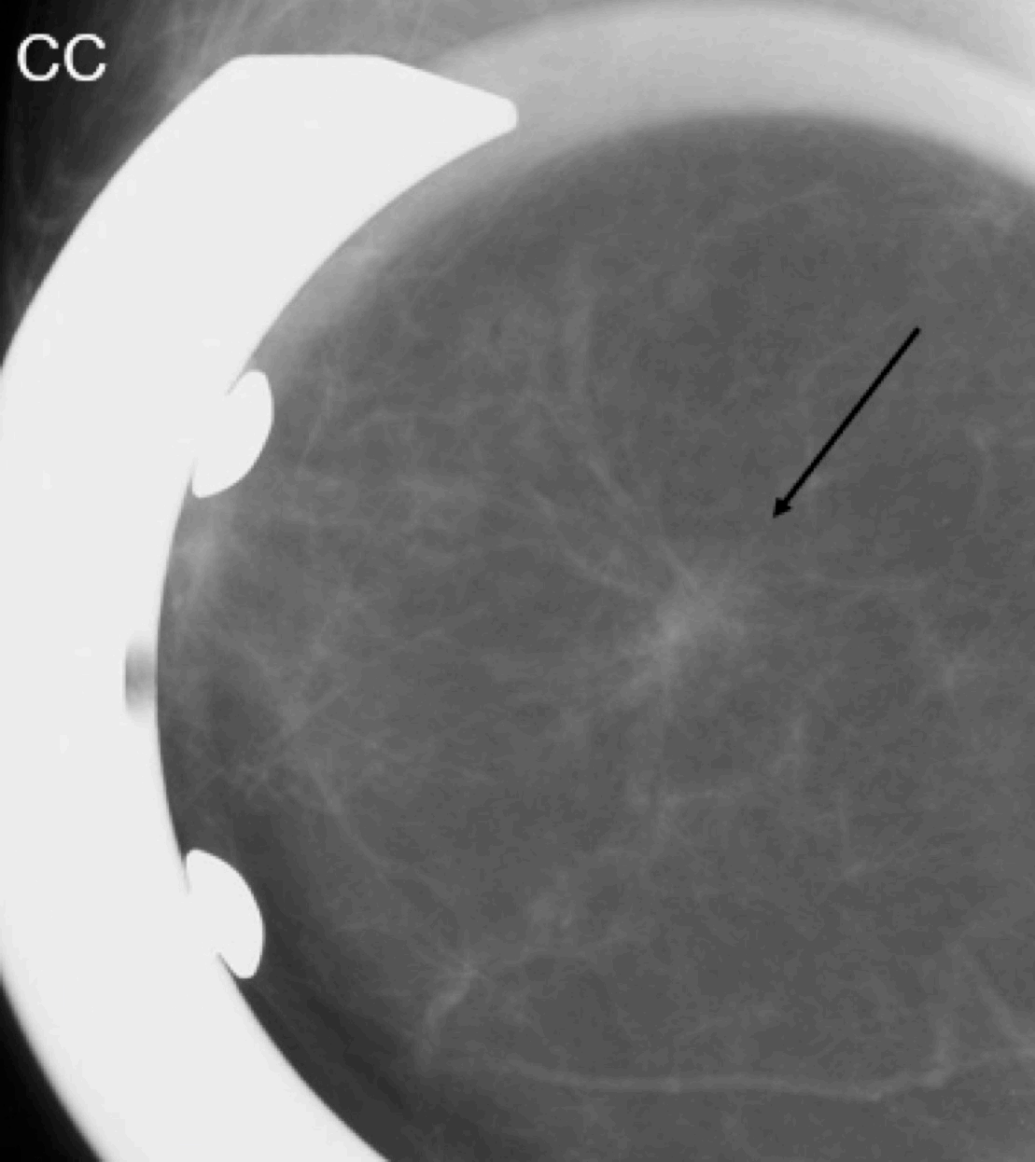
Mammographic spot CC view of an irregular spiculated mass with associated distortion.

**Figure 2.**
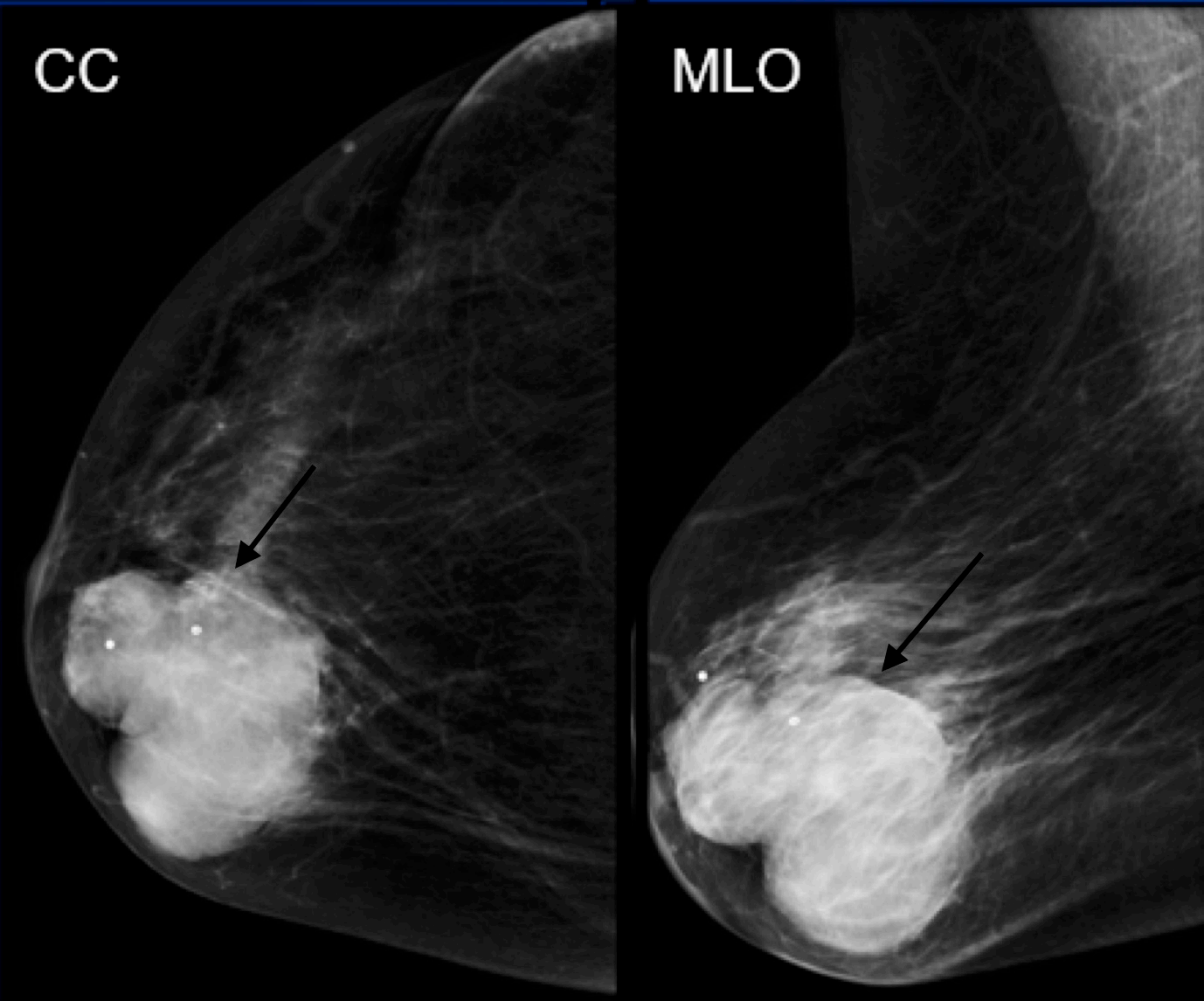
Mammographic CC and MLO views of a large oval circumscribed mass.

**Figure 3.**
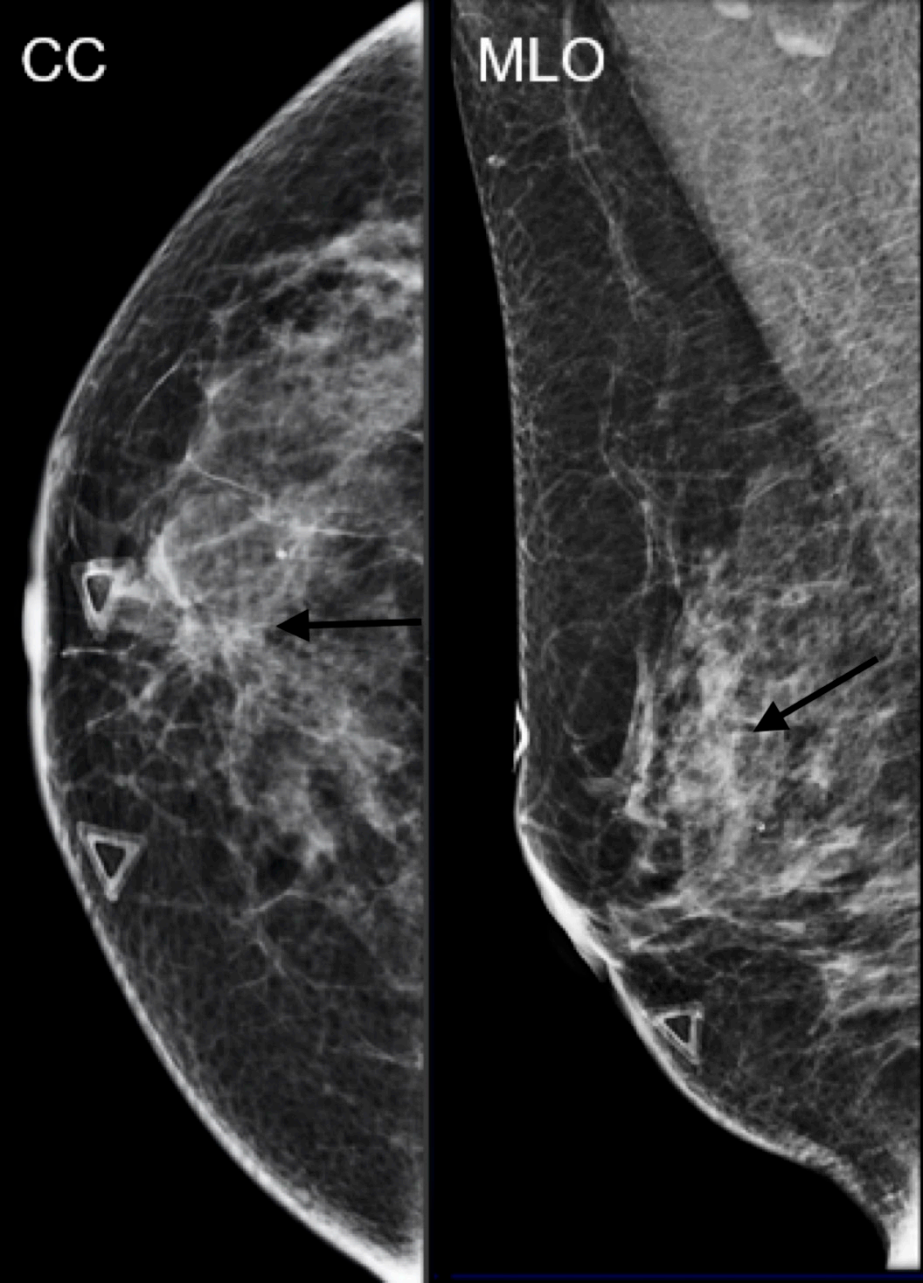
Mammographic CC and MLO views of an irregular indistinct mass.

**Figure 4.**
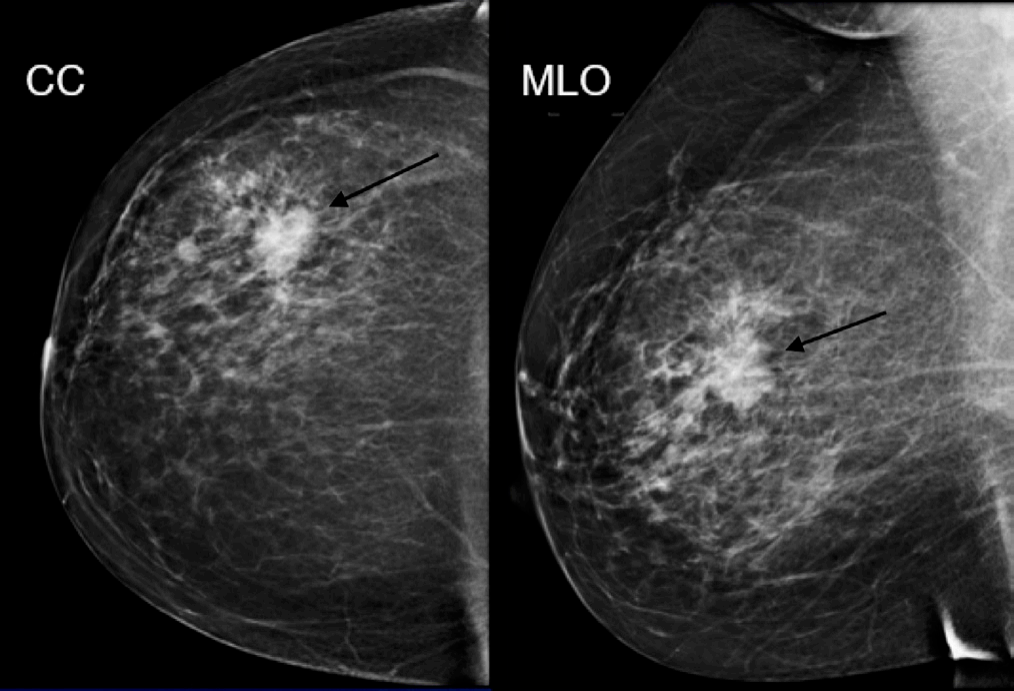
Mammographic CC and MLO views of an irregular spiculated mass. Pathology: Low-grade adenosquamous carcinoma

All seven cases had mammograms available for review. Mammographically, the majority presented as a suspicious irregular mass with either spiculated or indistinct margins (*n* = 5) (Figures 1–4). Only one patient presented as an oval circumscribed mass as seen in majority of the metaplastic literature (Figure 2).^1–4^ One patient presented as a focal asymmetry, which possibly represented a mass however imaging evaluation of this case was limited as only old mammograms without tomosynthesis were available for review.

Sonography

**Figure 5.**
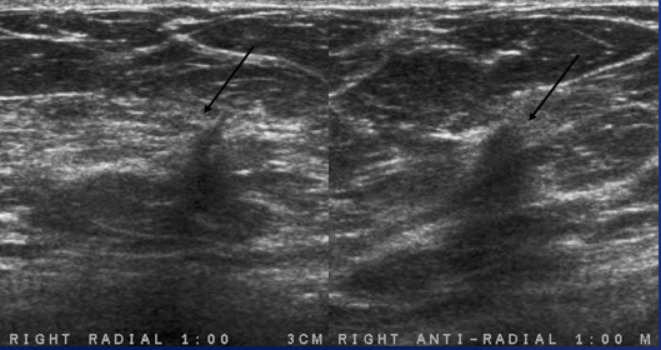
Ultrasound images in the right breast at 1:00, 3 cm from the nipple demonstrates an irregular hypoechoic mass with angular margins.

**Figure 6.**
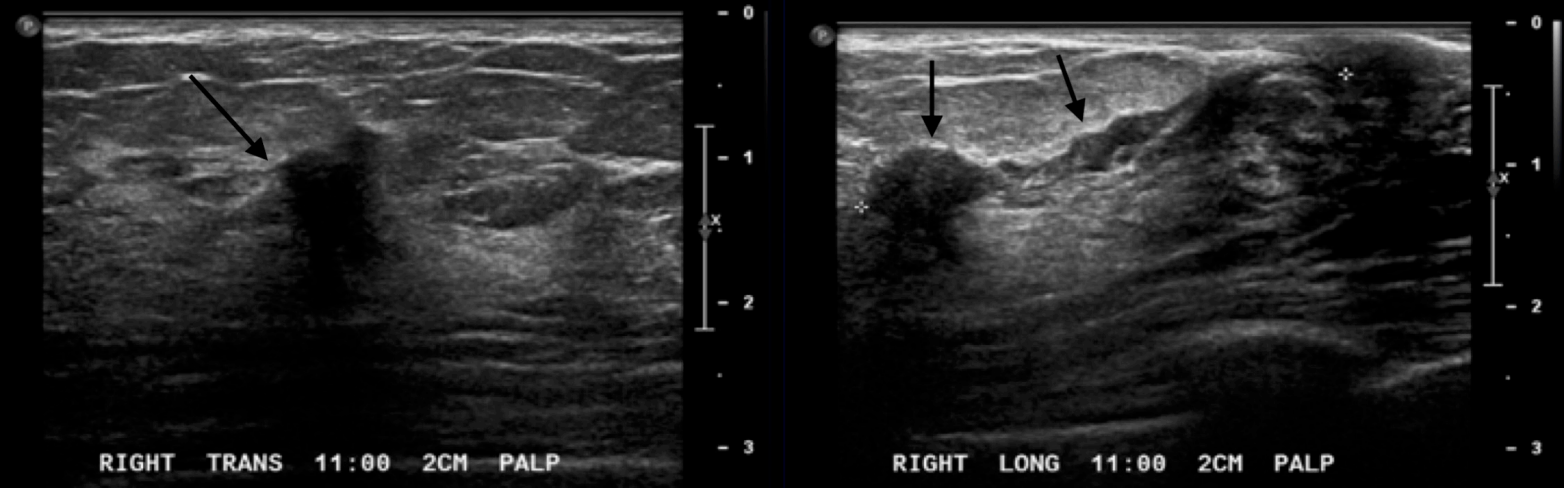
Ultrasound images in the right breast at 11:00, 2 cm from the nipple demonstrates an irregular indistinct hypoechoic mass with an associated dilated duct extending toward the nipple.

**Figure 7.**
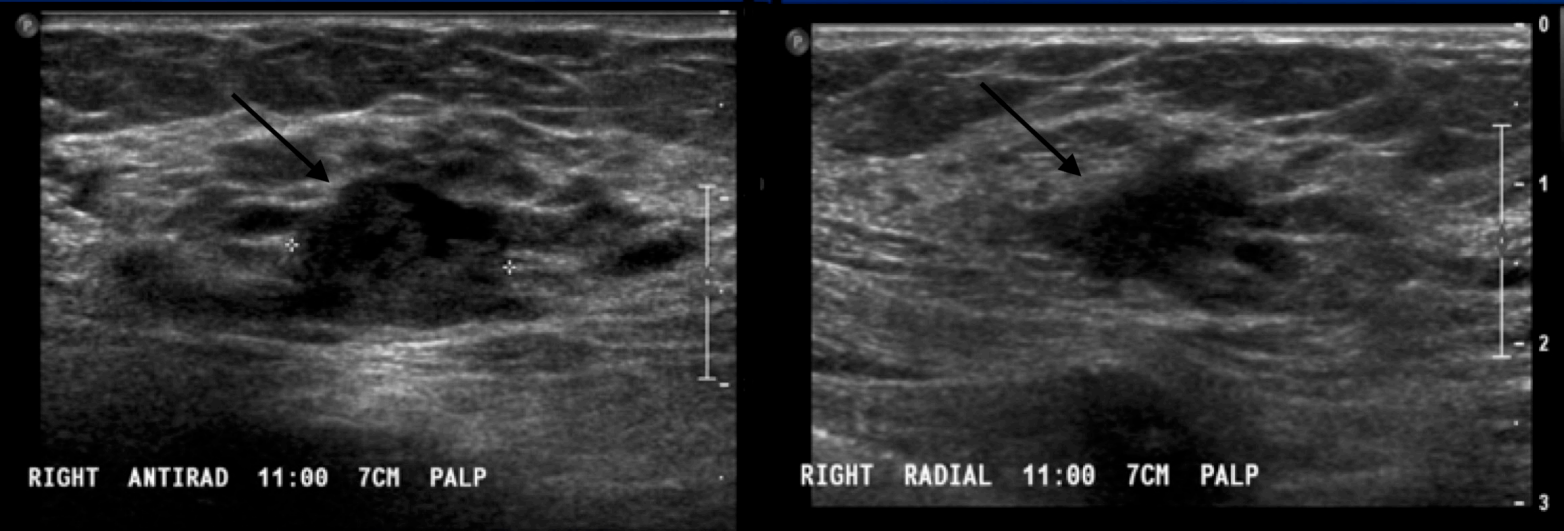
Ultrasound images in the right breast at 11:00, 7 cm from the nipple demonstrates an irregular indistinct hypoechoic mass.

**Figure 8.**
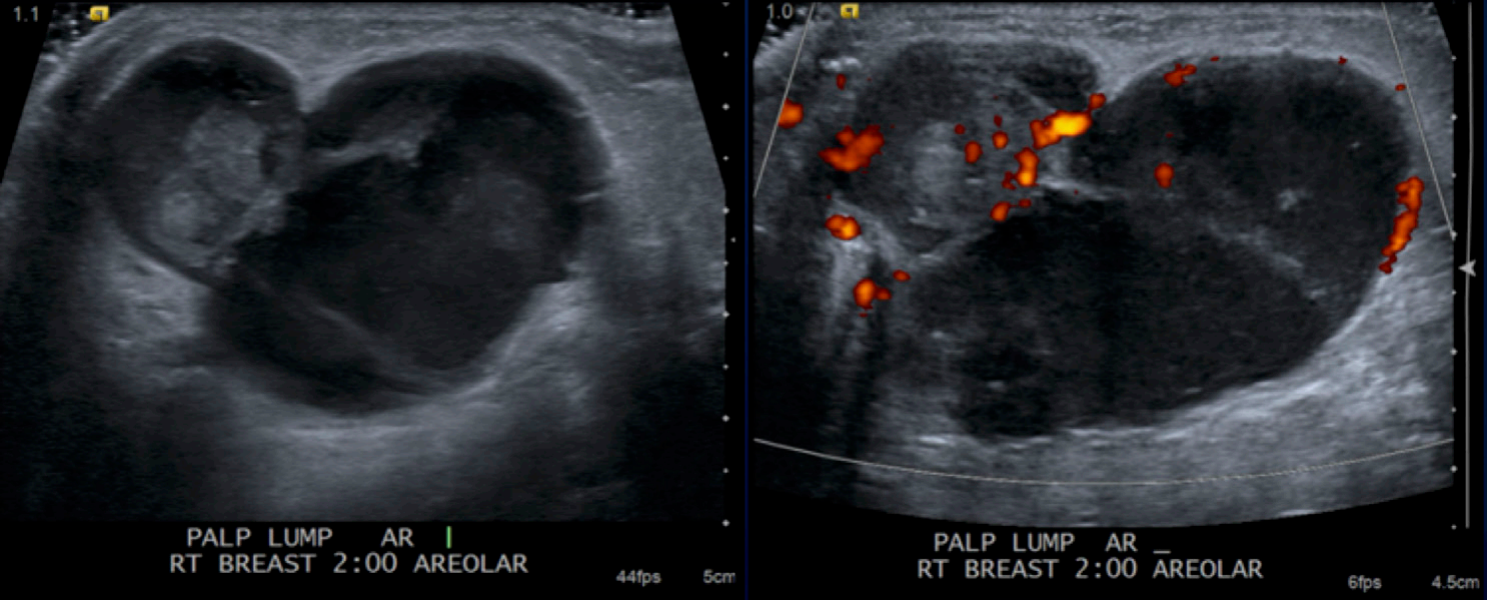
Ultrasound images in the right breast at 2:00, subareolar demonstrates a circumscribed mixed solid and cystic mass with increased internal vascularity.

All our cases were imaged with ultrasound and all demonstrated a mass. The majority presented as a suspicious irregular hypoechoic mass (*n* = 6) (Figures 5–7) with only one patient presenting as an oval circumscribed mass as seen in majority of the literature (Figure 8)^1–3^ Interestingly, one patient with an indistinct mass had an associated dilated duct extending toward the nipple which pathologically was determined to be low-grade adenosquamous carcinoma with adjacent ductal carcinoma *in situ* (Figure 6). The majority of patients had staging axillary ultrasounds performed (*n* = 5) with only one patient demonstrating an abnormal lymph node with cortical thickening. Fine needle aspiration biopsy of this node was negative for metastatic involvement.

#### MRI

**Figure 9.**
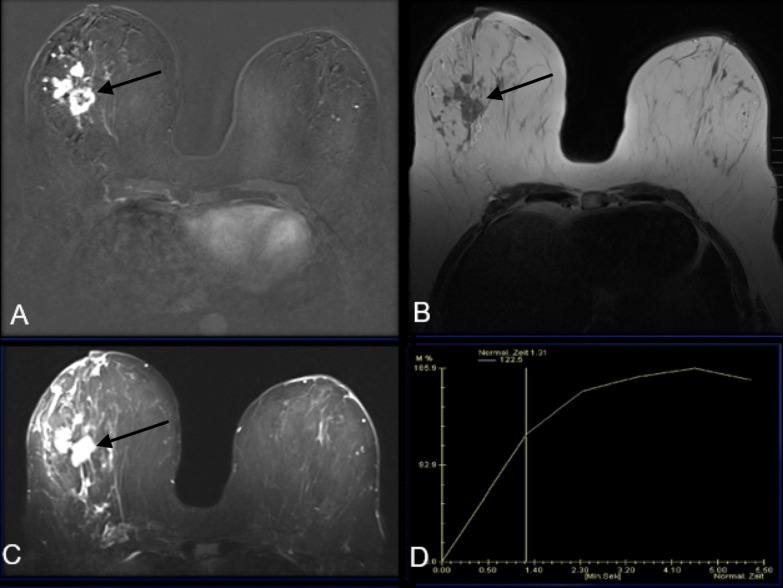
A- MRI subtraction post-contrast image demonstrates an irregular rim enhancing mass in the right outer middle depth breast. (**B**)- MRI *T_1_* weighted image demonstrates a T1 hypointense irregular mass (**C**)- MRI *T_2_* weighted image demonstrates a T2 hyperintense irregular mass (**D**)- MRI kinetic curve demonstrating Type 1 or persistent kinetics of this irregular mass

One of our adenosquamous cases had preoperative MRI available for review which demonstrated an irregular, rim enhancing, T1 hypointense, T2 hyperintense mass, similar to the reported metaplastic literature (Figure 9A, B and C).^8^ However, our case demonstrated benign Type 1 kinetics rather than the reported Type 2 and Type 3 kinetics in the literature (Figure 9D).^8^ No axillary or internal mammary lymphadenopathy was demonstrated.

Nuclear Medicine

**Figure 10.**
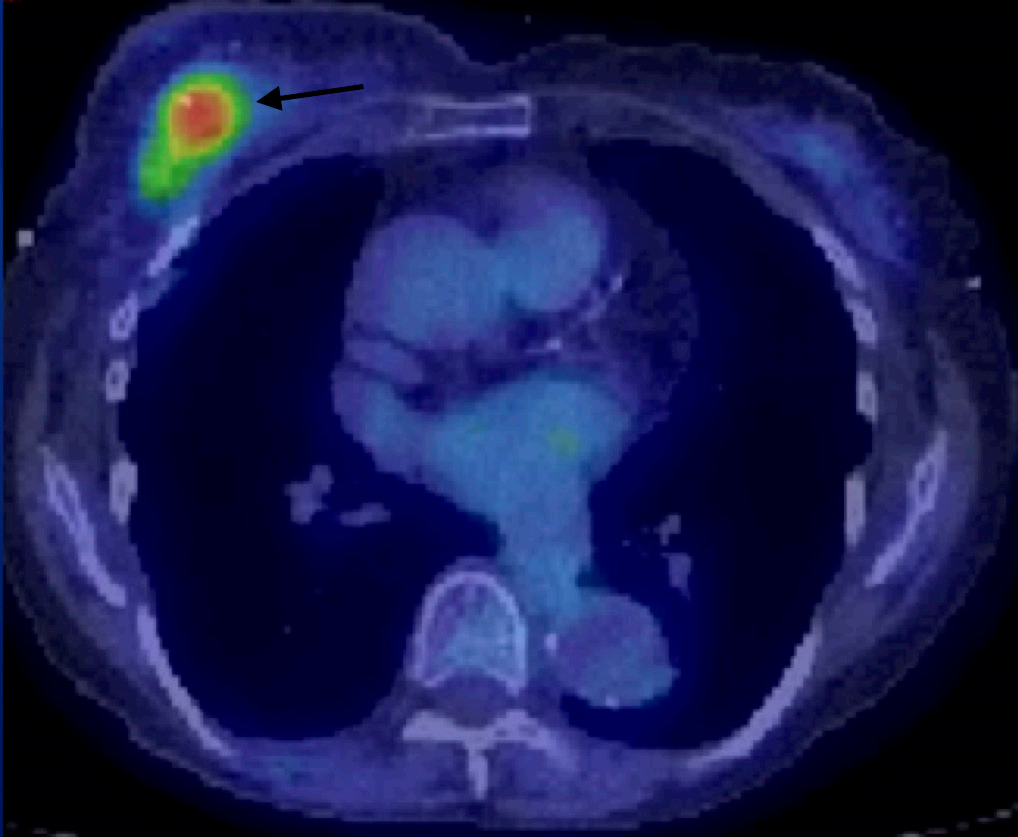


**Figure 11.**
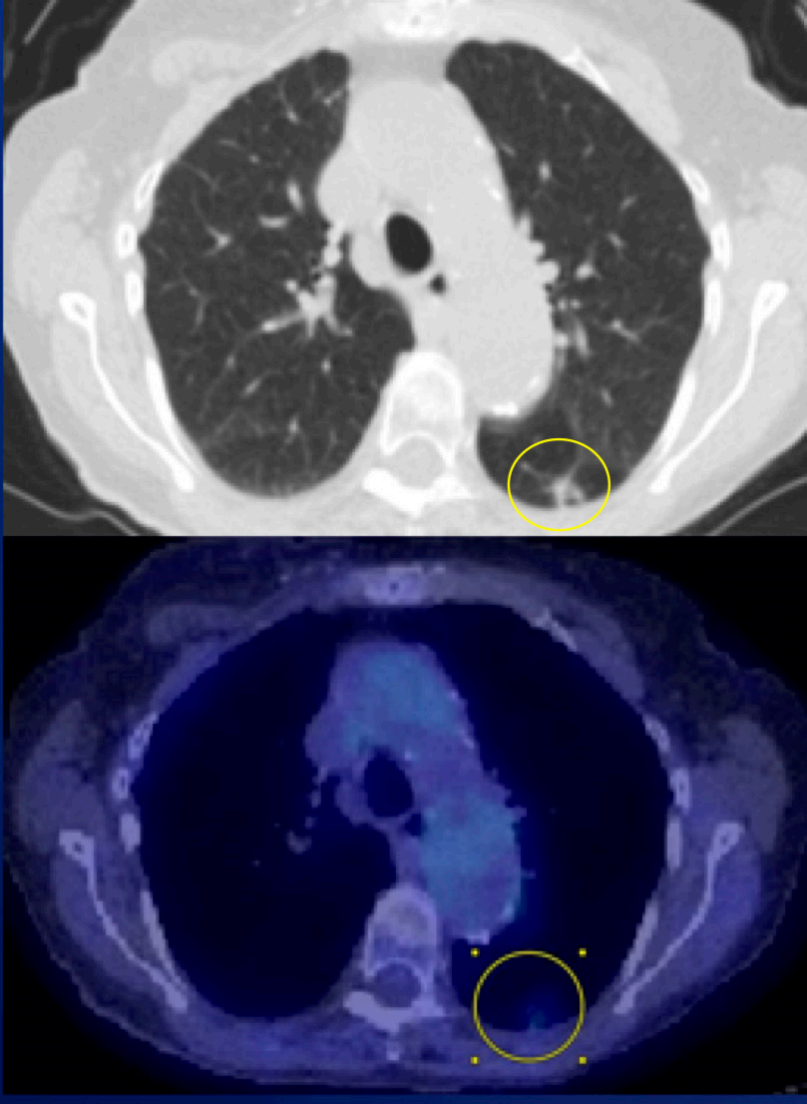


Two cases had FDG PET/CT available for review with one performed in the post-operative setting and the other case performed at time of disease recurrence a year after treatment. This one case with disease recurrence demonstrated FDG avid left breast masses as well as an enlarging FDG avid pulmonary nodule concerning for possible metastasis versus a secondary lung primary (Figures 10 and 11). She would later undergo pulmonary wedge resection with pathology positive for squamous cell carcinoma.

**Figure 12.**
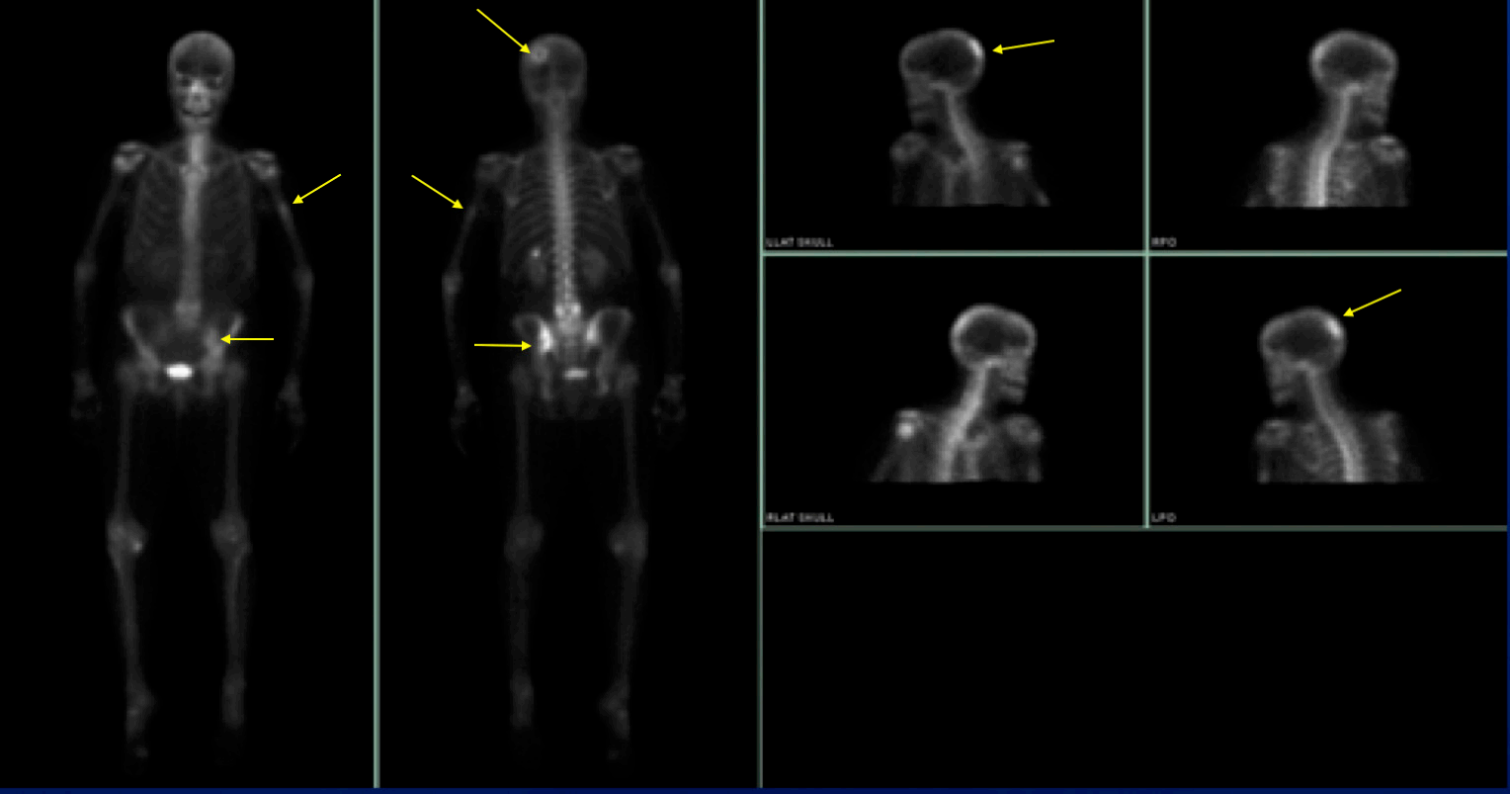


One case had a positive whole-body MDP bone scan available for review which demonstrated biopsy proven bony metastases. Abnormal radiotracer uptake was noted in the left parietal bone, left ileum, and left humerus (Figure 12). This patient was the only case presenting initially with high-grade pathology and metastases.

### Pathology characteristics

The majority of our cases presented as low grade (*n* = 6) similar to the reported literature. The only patient with disease recurrence initially presented as low grade and later immediate grade at recurrence. The only patient with biopsy proven metastasis was also the only case to initially present as a high-grade malignancy. Additional pathologic tumor associations included adenosquamous carcinoma with spindle cell features (*n* = 2), DCIS (*n* = 2) and complex sclerosis (*n* = 1).

ER/PR status as well as HER-2/neu status were performed in all cases. All cases were ER negative and PR negative. Majority of patients were HER-2/neu negative (*n* = 6) with only one patient HER-2/neu positive. This is consistent with the majority of metaplastic carcinoma as well as low-grade adenosquamous variant reported to be hormone receptor and HER-2/neu negative.^1,8,9^

### Clinical treatment and clinical outcomes

**Figure 13.**
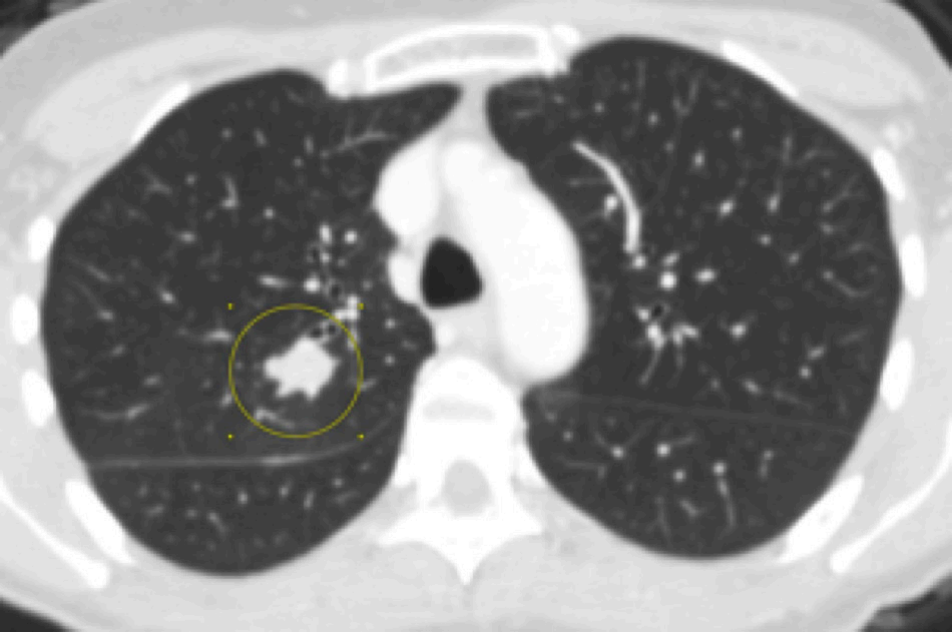


All seven cases underwent surgical treatment with 57% initially opting for mastectomy (*n* = 4) and 43% of patients initially receiving breast conservation surgery (*n* = 3). One patient would later undergo mastectomy after disease recurrence. Axillary lymph nodes were removed at time of surgery in 86% of patients (*n* = 6). Five patients had negative lymph nodes removed ranging from 1 up to 12 nodes. Only one patient had one of three nodes positive for involvement. Of seven cases, only one patient initially had biopsy proven metastatic disease with bony and pulmonary metastasis (Figures 12 and 13). Their left ileum bone biopsy was consistent with metaplastic carcinoma and new pulmonary masses measured up to 15 mm.

Only one patient received neoadjuvant chemotherapy, three patients received chemotherapy, and four patients received radiotherapy. Clinical outcomes was limited as follow-up was available in 57% of cases with 6–13 years of follow-up (*n* = 4). Only one known patient had biopsy-proven local recurrence with questionable pulmonary metastatic disease versus secondary lung primary. Follow-up was unavailable for 43% of cases (*n* = 3) as these patients decided to continue their treatment elsewhere due to geographical location from home.

## Representative cases

### Case 1

A 69-year-old female initially presented with questionable distortion on screening mammogram and called back for additional imaging. Diagnostic spot compression CC view demonstrates an irregular spiculated mass with distortion. Same-day diagnostic ultrasound images demonstrated an irregular hypoechoic mass with angulated margins. Pathology of this mass was positive for low-grade adenosquamous carcinoma.

## Case 2

A 82-year-old female initially presented with a palpable lump and focal pain. Diagnostic right breast mammogram with full-field CC and MLO views demonstrate a large oval circumscribed mass. Focused ultrasound in this region demonstrated a circumscribed mixed solid and cystic mass. Pathology was positive for low-grade adenosquamous carcinoma.

## Case 3

A 56-year-old female presents with palpable lumps. Diagnostic right breast mammogram demonstrates an irregular spiculated mass. Focused ultrasound in the right breast in the region of clinical concern demonstrates an irregular indistinct hypoechoic mass with an associated dilated duct extending towards the nipple. Pathology of this mass was positive for low-grade adenosquamous carcinoma.

## Case 4

A 47-year-old female presents as a palpable right breast lump. Diagnostic mammogram demonstrates an irregular spiculated mass. This patient also had a pre-operative MRI available for review which demonstrated an irregular large rim enhancing mass with associated non-mass enhancement and adjacent smaller masses in the right outer, middle depth breast. The dominant rim enhancing mass was T1 hypointense, T2 hyperintense, with persistent Type 1 kinetics. No enlarged axillary or internal mammary nodes were visualized. Pathology proven low-grade adenosquamous carcinoma.

## Case 5

A 84-year-old female with palpable lump initially demonstrated as an irregular indistinct mass both on mammogram and ultrasound. This patient would undergo lumpectomy with pathology proven low-grade adenosquamous carcinoma. One year later, this patient would demonstrate recurrent disease in the right breast on PET/CT as well as an FDG avid pulmonary nodule in the superior segment of the left lower lobe. She would undergo mastectomy. Pathology was positive for intermediate-grade adenosquamous carcinoma.

## Case 6

A 58-year-old female presented with a palpable lump. She was the only patient to initially present with pathology proven high-grade adenosquamous carcinoma, osseous as well as pulmonary metastasis.

Figures 12 and 13- Tc99m-MDP bone scan demonstrating biopsy proven bony metastatic disease within the left parietal, left humeral, and left ileum uptake. CT chest in the same patient with 1.5-cm right upper lobe pulmonary metastasis.

## Discussion

Given its rare presentation, discussion of the adenosquamous variant in the imaging literature has primarily been through case series of metaplastic breast cancer.^2,3^ The largest series in recent literature describing the mammographic and sonographic appearance of metaplastic breast cancer in 43 patients was performed by Yang et al in 2007.^1^ Metaplastic carcinoma has been reported to have a more benign mammographic and sonographic imaging appearance despite its typical high-grade, aggressive nature.^1–3^ The limited case reports and case series focusing on adenosquamous carcinoma have demonstrated no pathognomonic mammographic or sonographic imaging findings.^1–4^ Our cases of adenosquamous carcinoma demonstrated more classic suspicious mammographic and sonographic imaging features despite being low-grade tumors.

On MRI, metaplastic carcinoma typically appears as an irregular, spiculated mass.^8^ The largest series in recent literature detailing the MRI imaging features of metaplastic breast cancer in 12 cases was reported by Velasco et Al in 2005.^8^ Masses were typically T1 isointense to hypointense and T2 hyperintense with a necrotic component.^8^ To our knowledge, there are no reports of MRI imaging features focusing on the adenosquamous variant. Our one case available with MRI imaging demonstrated an irregular mass with similar imaging features as reported in the literature. The exception to our case was that this mass had benign Type 1 kinetics rather than Type 2 or Type 3 kinetics.^8^

**Figure 14.**
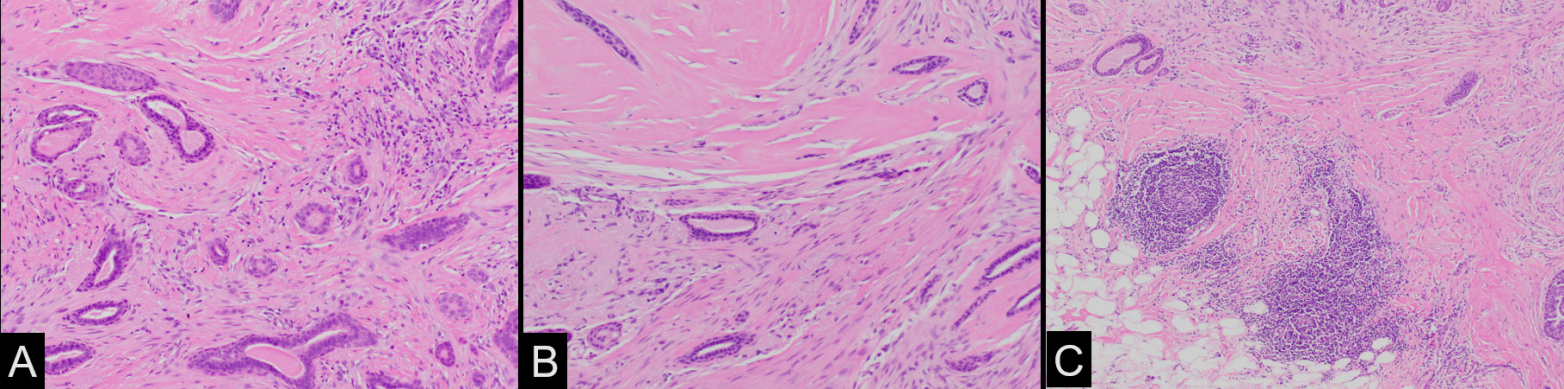
A. The tumor is composed of infiltrating glands which may be round or comma-shaped and atypical squamous cell nests. (Hematoxylin & Eosin, 200X) B. Compressed tumor glands are infiltrate either a cellular stroma or collagenized stroma. (Hematoxylin & Eosin, 200X) C. Peripheral lymphoid aggregates are often identified (Hematoxylin & Eosin, 100X)

Pathologically, metaplastic carcinomas are rare ductal carcinomas that undergo metaplasia into a non-glandular growth pattern.^3,4^ There are six histologic subtypes of tumors classified based on their epithelial and mesenchymal components.^1,9^ Adenosquamous is one of the rare subtypes typically presenting as a low-grade malignancy with an overall better prognosis.^5–7^ It is characterized by an admixture of infiltrating small, round to compressed and comma-shaped glandular structures and atypical squamous nests in a background of variably cellular stromal tissue.^5,7^ The periphery of the lesion often shows lymphoid aggregates.^10^ The infiltrating component blends subtly with normal background benign breast structures (Figure 14),^5,7^ often making delineation of the extent of lesion challenging.

All our cases were ER negative and PR negative. The majority of our cases were HER-2/neu negative (*n* = 6) with only one patient HER-2/neu positive. This is consistent with the majority of metaplastic carcinoma, as well as low-grade adenosquamous variant, reported to be hormone receptor and HER-2/neu negative.^1,2,6,8,9^ There have been prior pathology reports of low-grade adenosquamous carcinoma demonstrating HER-2/neu positivity.^7^ Typically, tumors with a negative hormone receptor status are associated with a worse prognosis.^7^ There is a dissociation between the expected prognosis based on expression of biochemical markers and the observed better prognosis in adenosquamous carcinoma.^7^ This suggests that histological classification of mammary carcinoma itself is an important prognostic variable.

There is currently no consensus on the optimal treatment for adenosquamous carcinoma. However, the risk of local recurrence has led to aggressive local treatment. Patients with adenosquamous carcinoma may be good candidates for breast conservation therapy.^5^ There is a very low incidence of axillary lymph node involvement and metastatic disease.^4,5,7,9^

## Conclusion

Adenosquamous carcinoma is a rare subtype of metaplastic breast carcinoma. Metaplastic carcinoma of the breast accounts for <1% of invasive breast malignancy.^5,6^ Similar to other types of metaplastic carcinoma, adenosquamous carcinoma has been reported to typically clinically present as a palpable mass as seen in the majority of our patients.^2–5,7^ Patients typically present over the age of 50 as seen in our cases.^3,4,8^ Unlike other subtypes of metaplastic breast carcinoma, the adenosquamous variant has a favorable prognosis.^5^ Most cases are reported as low-grade malignancy with locally invasive growth. Metaplastic carcinomas are reported to have a more benign mammographic and sonographic imaging appearance despite its typically high grade aggressive nature.^2–4^ Our cases of adenosquamous carcinoma demonstrated more classic suspicious imaging features despite majority being low-grade tumors. Although there are no specific imaging features, it is important to keep metaplastic carcinoma in the differential with the adenosquamous variant having more suspicious imaging features despite better outcomes.

## Learning points

- Adenosquamous carcinoma is a rare subtype of metaplastic breast carcinoma.
- In general, metaplastic carcinoma is reported to have more a more benign imaging appearance despite typically presenting as a palpable, high-grade, aggressive malignancy. Our cases demonstrated predominantly suspicious imaging features despite majority being a low-grade, less aggressive malignancy.
- Adenosquamous carcinoma is commonly steroid receptor negative despite most being low-grade malignancies suggesting that histologic classification itself is an important prognostic variable.

## References

[1] Yang WT, Hennessy B, Broglio K, Mills C, Sneige N, Davis WG, et al. Imaging differences in metaplastic and invasive ductal carcinomas of the breast. AJR Am J Roentgenol 2007; 189: 1288–93. doi: 10.2214/AJR.07.2056 18029860

[2] Park JM, Han BK, Moon WK, Choe YH, Ahn SH, Gong G. Metaplastic carcinoma of the breast: mammographic and sonographic findings. J Clin Ultrasound 2000; 28: 179–86. doi: 10.1002/(SICI)1097-0096(200005)28:4<179::AID-JCU5>3.0.CO;2-Y 10751739

[3] Günhan-Bilgen I, Memiş A, Ustün EE, Zekioglu O, Ozdemir N. Metaplastic carcinoma of the breast: clinical, mammographic, and sonographic findings with histopathologic correlation. AJR Am J Roentgenol 2002; 178: 1421–5. doi: 10.2214/ajr.178.6.1781421 12034610

[4] Patterson SK, Tworek JA, Roubidoux MA, Helvie MA, Oberman HA. Metaplastic carcinoma of the breast: mammographic appearance with pathologic correlation. American Journal of Roentgenology 1997; 169: 709–12. doi: 10.2214/ajr.169.3.9275883 9275883

[5] Rosen PP, Ernsberger D. Low-Grade adenosquamous carcinoma. A variant of metaplastic mammary carcinoma. Am J Surg Pathol 1987; 11: 351–8. doi: 10.1097/00000478-198705000-00003 3578645

[6] Barnes PJ, Boutilier R, Chiasson D, Rayson D. Metaplastic breast carcinoma: clinical-pathologic characteristics and HER2/neu expression. Breast Cancer Res Treat 2005; 91: 173–8. doi: 10.1007/s10549-004-7260-y 15868445

[7] Drudis T, Arroyo C, Van Hoeven K, Cordon-Cardo C, Rosen PP. The pathology of low-grade adenosquamous carcinoma of the breast. An immunohistochemical study. Pathol Annu 1994; 29: 191–7. 7936747

[8] Velasco M, Santamaría G, Ganau S, Farrús B, Zanón G, Romagosa C, et al. Mri of metaplastic carcinoma of the breast. AJR Am J Roentgenol 2005; 184: 1274–8. doi: 10.2214/ajr.184.4.01841274 15788609

[9] Tse GM, Tan PH, Putti TC, Lui PCW, Chaiwun B, Law BKB. Metaplastic carcinoma of the breast: a clinicopathological review. J Clin Pathol 2006; 59: 1079–83. doi: 10.1136/jcp.2005.030536 16467167 PMC1861754

[10] Soo K, Tan PH. Low-Grade adenosquamous carcinoma of the breast. J Clin Pathol 2013; 66: 506–11. doi: 10.1136/jclinpath-2012-201084 23268316

